# Occupational exposures of firefighting and prostate cancer risk in the Norwegian Fire Departments Cohort

**DOI:** 10.5271/sjweh.4202

**Published:** 2025-03-01

**Authors:** Niki Marjerrison, Tom K Grimsrud, Johnni Hansen, Jan Ivar Martinsen, academic degree,, Karl-Christian Nordby, Raymond Olsen, Jo S Stenehjem, Marit B Veierød, Kristina Kjærheim

**Affiliations:** 1 Oslo Centre for Biostatistics and Epidemiology, Department of Biostatistics, Institute of Basic Medical Sciences, University of Oslo, Oslo, Norway.; 2 Department of Research, Cancer Registry of Norway, Norwegian Institute of Public Health, Oslo, Norway.; 3 Danish Cancer Society Research Center, Copenhagen, Denmark.; 4 National Institute of Occupational Health, Oslo, Norway.

**Keywords:** carcinogen, cohort study, occupational epidemiology

## Abstract

**Objectives:**

Excess incidence of prostate cancer (PC) is frequently observed among firefighters; however, the association with specific occupational exposures of firefighting, as well as the influence of a medical surveillance bias, remains unclear. Our aim was to study PC risk within a firefighter cohort, applying indicators of exposures.

**Methods:**

We used indicators of various firefighting exposures to examine PC risk among men in the Norwegian Fire Departments Cohort (N=4251). Incident PC cases, including clinical characteristics, were obtained from the Cancer Registry of Norway (1960–2021). Cox regression was used to estimate hazard ratios (HR) by cumulative exposure in tertiles (reference: lowest) for all, aggressive, and indolent PC, with adjustment for age and birth cohort. The cumulative incidence of PC across birth cohorts and diagnostic periods was examined.

**Results:**

No clear associations emerged for any of the exposure indicators, although we observed an HR of 1.31 [95% confidence interval (CI) 0.63–2.72] for aggressive PC in the highest tertile of fire exposure score and 1.31 (95% CI 0.60–2.89) for indolent PC in the highest tertile of inhalation score. Assessment of cumulative incidence demonstrated a greater number of diagnoses at younger ages after 1990, particularly for indolent and unclassifiable PC.

**Conclusions:**

We found little support for an association between firefighting exposures and PC risk. However, our study had few cases in analyses by clinical stage. Challenges in studies of firefighters’ PC risk remain, including difficulties in exposure characterization and the unclear magnitude of a medical surveillance bias.

Studies of firefighters’ cancer risk have frequently observed elevated prostate cancer (PC) incidence compared to the general population, while PC mortality is often at unity (1–4). Likewise, a previous study of the Norwegian Fire Departments Cohort observed elevated PC incidence and PC mortality at unity compared to the general population (5).

Firefighters can be exposed to numerous carcinogens through their work, such as polycyclic aromatic hydrocarbons (PAH), polychlorinated biphenyls, 1,3-butadiene, 2,3,7,8-tetrachlorodibenzo-*para*-dioxin, asbestos, and diesel exhaust, among others (1). However, given the nature of their work, measurements of occupational exposures for use in epidemiological studies are widely unavailable. Further, understanding of PC etiology is limited, and no agent, occupational or otherwise, has yet been identified as a prostate carcinogen with sufficient evidence in humans (6).

Some studies of firefighters’ cancer risk have developed exposure indicators, such as number and type of fires attended, to investigate exposure–response associations (7, 8). In an Australian cohort, an increased risk of PC with increasing number of fires attended was observed (8), while in a US cohort, no evidence of an association with number of fires attended was observed (7).

Interpreting findings of firefighters’ elevated PC risk is also challenging because PC incidence has been influenced by prostate-specific antigen (PSA) testing, which led to a steep increase in the PC incidence rate in Norway from around 1990 to the mid-2000s (9–11). Firefighters in Norway undergo regular health surveillance but there is no general or occupational PSA screening program, and the influence of opportunistic PSA testing on findings of elevated incidence is unclear. Lending some support to the hypothesis of a contribution from PSA testing, Norwegian firefighters have been observed to be younger and have better prognostic markers at diagnosis compared to the general population (12). In a recent evaluation of the carcinogenicity of firefighting, the International Agency for Research on Cancer deemed the evidence for the overall positive association with PC “limited” because a medical surveillance bias could not be reasonably excluded (1, 13, 14).

Using detailed work histories, newly developed indicators of exposure to fire smoke and diesel exhaust (15) and clinical characteristics of PC cases, we investigated the associations between indicators of firefighting exposures and PC risk within the Norwegian Fire Departments Cohort.

## Methods

### Study cohort

The Norwegian Fire Departments Cohort has been described previously (16). Fifteen fire departments in Norway, including many of the largest departments, registered all employees active in 1950–2018 with the following: birth date; personal identification number; vocational training; department and station(s) at which they worked; and employment history (job titles, time periods and employment percentage for each position).

The cohort includes 4627 people. We excluded women (N=291), men who died before start of follow-up (1 January 1960; N=30), and men who began employment in 2019 (N=11) due to incomplete registration after 2018. Employees registered only in 0% positions (N=20) or who lacked a job title (N=24) were excluded as analyses were based on active and detailed employment history. Thus, 4251 men were eligible for the study, including 14 904 work periods.

### Exposure assessment

Development of the exposure indicators has been described previously (15). The process was aided by the project reference group, comprising representatives from firefighters’ unions, employers’ organizations, the Norwegian Directorate for Civil Protection, Norwegian Labour Inspection Authority, Norwegian Cancer Society, and Norwegian Firefighters Fight Cancer.

Six indicators are set out in table 1: (i) duration of employment, (ii) duration of fire-exposed employment, (iii) number of fires attended, (iv) fire exposure score, (v) inhalation score and (vi) diesel exhaust exposure score.

**Table 1:** Overview of exposure indicators showing the exposures they are intended to reflect, the period they were available for and the year-specific data they are based on. Previously published in Marjerrison et al (15).

Indicator	Exposure	Period	Data used
Duration of employment	General exposure of employment at a fire department	1913–2018	All periods of active employment
Duration of fire-exposed employment	General exposure of active firefighting duties	1913–2018	All periods of active employment Firefighting exposure potential for category of job held
Number of fires attended	Fire, smoke, and diesel exhaust exposure at the fire scene	1940–2015	All periods of active employment Firefighting exposure potential for category of job held Annual number of fires (86% structural)
Fire exposure score	Fire and smoke exposure via inhalation, based on number of fires attended and considering respiratory protection	1940–2015	All periods of active employment Firefighting exposure potential for category of job held Type and use of SCBA (negative pressure, manual positive pressure, or automatic positive pressure)
Inhalation score	Fire and smoke exposure via inhalation, based only on respiratory protection	1940–2015	All periods of active employment Firefighting exposure potential for category of job held Type and use of SCBA (negative pressure, manual positive pressure, or automatic positive pressure)
Diesel exhaust exposure score	Diesel exhaust exposure at the fire station	1940–2015	All periods of active employment Number of diesel vehicles Station design (reflecting possibility for air passage from the vehicle bay) Use of an exhaust removal system

*Duration of employment* reflected the general exposure of employment at a fire department and was calculated for the entire period over which employment data spanned (1913–2018) by accruing each person’s cumulative employment duration.

*Duration of fire-exposed employment* reflected the general exposure of active firefighting, also for the period 1913–2018. Job titles (N=184) in the cohort were grouped into categories with a factor reflecting their potential for exposure to live firefighting, as follows: fully exposed (factor 1); partly exposed (factor 0.5); non-exposed (factor 0); and other exposed (factor 0.5), where there was no potential for firefighting exposure but there was potential for similar exposures (15). For each person, their duration of fire-exposed employment was summed cumulatively according to each year and percentage of their active employment multiplied by the factor reflecting the category-specific exposure potential for the job title held.

*Number of fires attended* reflected exposure from fire, smoke, and diesel exhaust at the fire scene for the period 1940–2015. The annual number of fire-related responses attended by fire departments were provided by the Norwegian Directorate for Civil Protection for the period 1986–2015. For each department, the average number of fire runs in the period 1986–1995 was extrapolated to 1940 to estimate exposures also for the high number of employees active from 1940. The department’s annual sum of fire responses was divided by the number of teams active at each department to determine the number of fires attended by a single firefighter in each year.

*Fire exposure score* reflected exposure from fire, smoke, and diesel exhaust at the fire scene occurring via inhalation for the period 1940–2015. In addition to the number of fires attended, this indicator considered changes in the type and use of personal protective equipment (PPE), based on fire departments’ responses to a questionnaire on working conditions (17). The questionnaire recorded the types of self-contained breathing apparatuses (SCBA) used during the three phases of structural firefighting (smoke diving, knockdown, and overhaul). The influence on exposure according to SCBA type and use was determined according to the assigned protection factors during workplace-simulated use (18) and the reported use at Norwegian fire departments. Thus, for exposure from smoke diving, this corresponded to a protection factor of 0.01 for positive-pressure SCBA compared to negative-pressure SCBA, and 0.5 for manual positive-pressure SCBA. For exposure during knockdown and overhaul, the factor for positive-pressure SCBA was 0.05 due to expected less-than-ideal use. The number of fires attended per year was multiplied by these SCBA protection factors to determine the fire exposure score for each department in each year.

*Inhalation score* also reflected fire and smoke exposure via inhalation, for the period 1940–2015 but used information on respiratory protection use only as there was some unexplained variation in the fire statistics. Therefore, for each year and department, a score was determined using the aforementioned protection factors pertaining to SCBA use.

Each individual’s number of fires attended, fire exposure score and inhalation score were summed cumulatively according to their years and percentages of active employment, also multiplied by the factor reflecting potential for exposure to live firefighting in the category of job title held.

*Diesel exhaust exposure score* reflected diesel exhaust exposure at the fire station for the period 1940–2015. We estimated the combined relative intensity of exposure from diesel vehicles in the fire station garage as well as office, sleeping and living quarters based on fire departments’ responses in the aforementioned questionnaire (17). For garage exposure, the number of diesel vehicles in operation at each station was multiplied by a factor of 0.05 if a station had an exhaust removal system and by 1 if there was none. For office, sleeping and living quarters, the number of diesel vehicles was multiplied by a factor of 1 if there was certain free air passage from the garage, 0.5 for partial passage, and 0 if there was no passage or no sleeping or living quarters. Each individual’s diesel exhaust exposure score was summed cumulatively according to each year and percentage of active employment.

### Follow-up

The cohort was linked to national registries using Norwegian personal identification numbers for the period 1 January 1960–31 December 2021. Date of emigration was obtained from the Norwegian Population Register; date of death from the Cause of Death Registry; and date and diagnosis of cancer from the Cancer Registry of Norway (CRN) according to the International Classification of Diseases (10^th^ revision [ICD-10]). The CRN also provided information on summary stage at diagnosis, defined as localized, regional (regional lymph node metastasis or invasion of neighboring tissue), and distant (non-regional lymph node or organ metastasis) for 1960–2021. From 1 January 1994, the CRN provided data on clinical stage at diagnosis according to the Tumor-Node-Metastasis (TNM) Classification of Malignant Tumors (19) and, from 1 January 2004, on Gleason score (20).

PC cases were defined as malignant neoplasms of the prostate (ICD-10 C61). Aggressive PC cases were those having any of the following at diagnosis: summary stage reflecting regional or distant metastasis; clinical category of T4 (large tumor, locally advanced), N1 (lymph node involvement), or M1 (distant metastasis); or Gleason score ≥8 (21). Indolent PC cases were defined as localized malignancies according to summary stage (from 1960) and TNM classification (from 1994), and Gleason score <8 (from 2004). Unclassifiable PC cases were those that had no aggressive characteristics but which were missing information for at least one variable that ensured they were indolent. Indolent and unclassifiable PC were also grouped as “non-aggressive” PC. A previous study of Norwegian PC patients diagnosed with unknown metastatic status indicated that few patients in this group had distant metastases (22). Thus, we expect that our group of non-aggressive PC primarily reflected non-metastatic PC.

We conducted analyses for all PC combined and by clinical stage. Analysis by clinical stage was done to discern the effect of PSA testing by restricting analyses to aggressive PC, which would presumably have been detected irrespective of PSA testing opportunity, and isolating indolent and non-aggressive PC, which may have been detected through increased health surveillance.

### Statistical analysis

Cox proportional hazard regression with age as the timescale, stratified by 10-year birth cohort (≤1939, 1940–1949, …, ≥2000), was used to estimate hazard ratios [HR; 95% confidence intervals (CI)] for the association between firefighting exposures and PC risk. The six exposure variables were time-dependent and updated for each individual during follow-up. Stratification by birth cohort was intended to capture changes in PSA testing opportunity at different time periods and ages. For each individual, follow-up began at the age at the latter of 1 January 1960 or start of first employment, and continued until the age at the first of PC diagnosis, emigration, death, or 31 December 2021.

For analysis of all PC, exposures were categorized into tertiles with cut points at the nearest whole number for an approximately even distribution of cases (reference: lowest tertile). Analyses by clinical stage used the same cut points as for all PC combined. For duration of employment and fire-exposed employment, analyses were conducted with alternative cut points at ≤14 (reference), 15–24, and ≥25 years. Analyses with exposures lagged 10 and 15 years were conducted to account for a latency period between exposure and cancer diagnosis. Trend tests were performed by modelling ordinal variables as continuous.

Exposure scores using the indicators began in 1940; therefore, those who began employment before 1940 (N=228, 5%) were excluded from corresponding analyses. Employees at one station that did not provide information on diesel vehicles (N=84, 2%) were excluded from analyses of the diesel exhaust exposure score.

The following sensitivity analyses were conducted: additional adjustment for the healthy worker survivor effect (HWSE) (23, 24) using employment status per 31 December 2018 (still employed, stopped working/died at age <60 years, or stopped working ≥60 years); and restricting analysis to first primary PC (25).

Cumulative incidence functions were calculated with aggressive PC as the main outcome, treating non-aggressive PC and death as competing events. Cumulative incidence curves were plotted with age and calendar year as the time scale, according to birth cohort, as well as with age as the time scale according to follow-up periods.

All statistical analyses were performed using Stata V.18 (Stata Corp, College Station, TX, USA). The stcompet package was used to calculate cumulative incidence functions (26).

## Results

The 4251 men in our study accrued 123 835 person-years of follow-up. There were 268 PC cases (239 first primary), with 61 classified as aggressive (50 first primary) and 69 as indolent (65 first primary; table 2). The remaining 138 cases were unclassifiable; combined with indolent PC, this amounted to 207 non-aggressive cases. Mean age at diagnosis was 69.4 years for all PC cases, 72.1 years for aggressive PC, and 68.1 for indolent PC. Overall, 84% of all PC cases were diagnosed in 1994 or later (when TNM classification was included in the CRN). For aggressive PC, this proportion was 84%; for indolent PC, 55%; and for non-aggressive PC, 84%.

**Table 2:** Characteristics of the study sample from the Norwegian Fire Departments Cohort. Follow-up from 1 January 1960 to 31 December 2021. [PC=prostate cancer; SD=standard deviation].

		Total			All PC ^a^			Aggressive PC ^b^			Indolent PC ^c^			Non-aggressive PC ^d^	
		N (%)	Mean (SD)		N (%)	Mean (SD)		N (%)	Mean (SD)		N (%)	Mean (SD)		N (%)	Mean (SD)
		4251 (100)			268 (6.3)			61 (1.4)			69 (1.6)			207 (4.9)	
Birth year			1953 (24.7)			1937 (16.6)			1935 (16.9)			1933 (19.0)			1938 (16.5)
Age at start of follow-up (years)			31.4 (9.9)			32.8 (10.4)			34.1 (10.7)			35.8 (11.4)			32.4 (10.2)
Duration of follow-up (year)			29.1 (15.6)			36.6 (11.0)			38.0 (13.6)			32.4 (9.3)			36.2 (10.2)
Age at end of follow-up/diagnosis (years)			60.0 (16.4)			69.4 (9.3)			72.1 (10.6)			68.1 (6.7)			68.5 (8.7)
Year of diagnosis						2007 (12.0)			2007 (12.5)			2001 (15.2)			2006 (11.9)
Employment															
	Year of first employment		1981.0 (25.2)			1965.2 (17.7)			1963.0 (19.4)			1961.0 (18.8)			1965.8 (17.2)
	Age at first employment (years)		27.9 (6.6)			28.0 (7.0)			28.2 (7.8)			28.3 (5.2)			27.9 (6.8)
	Duration of employment (years)		20.5 (13.1)			28.3 (10.3)			27.0 (11.6)			28.1 (9.5)			28.6 (9.9)

^a^ International Classification of Diseases 10^th^ revision code C61. ^b^ Aggressive PC defined as any of the following at diagnosis: summary stage reflecting regional lymph node or distant metastasis; TNM categories T4, N1 or M1 (from 1994); or a Gleason score ≥8 (from 2004). ^c^ Indolent PC defined as the following at diagnosis: local malignancy according to summary stage (from 1960) and TNM category (from 1994), and a Gleason score <8 (from 2004). ^d^ All PC that were not defined as aggressive (above).

Lagging exposures did not contribute to essential differences; thus, the results below describe results with no lagging unless stated otherwise.

With duration of employment and duration of fire-exposed employment, for all PC combined, HR were in the range 0.97–1.11 for the middle and highest tertile compared to the lowest (table 3). For aggressive PC, the corresponding HR were 0.59–1.00. For non-aggressive PC, for the middle tertile of duration of employment, we observed an HR of 1.52 (95% CI 1.07–2.15) with a 10-year lag (P-trend 0.35) and 1.46 (95% CI 1.01–2.11) with a 15-year lag (P-trend 0.29). Analyses with alternative cut points (at 15 and 25 years) did not essentially change the results (results not shown).

**Table 3:** Exposure to occupational firefighting and prostate cancer (PC) risk among men in the Norwegian Fire Departments Cohort. Cox regression with age as the timescale, stratified by 10-year birth cohort. Follow-up from 1 January 1960 to 31 December 2021. [HR=hazard ratio; CI=confidence interval; Ref=reference].

Exposure			All PC^a^			Aggressive PC^b^			Indolent PC^c^			Non-aggressive PC^d^	
			Cases	HR (95% CI)		Cases	HR (95% CI)		Cases	HR (95% CI)		Cases	HR (95% CI)
Duration of employment (n=4251)													
	No lag												
		Tertile 1	89	1.00 (Ref)		25	1.00 (Ref)		23	1.00 (Ref)		64	1.00 (Ref)
		Tertile 2	92	1.07 (0.79–1.44)		14	0.61 (0.31–1.19)		31	1.29 (0.75–2.22)		78	1.24 (0.88–1.73)
		Tertile 3	87	1.11 (0.81–1.51)		22	1.00 (0.55–1.83)		15	0.65 (0.34–1.26)		65	1.15 (0.80–1.64)
		P-trend*		0.51			0.97			0.25			0.45
	Lag 10 years												
		Tertile 1	93	1.00 (Ref)		25	1.00 (Ref)		22	1.00 (Ref)		68	1.00 (Ref)
		Tertile 2	95	1.29 (0.94–1.75)		13	0.65 (0.32–1.32)		32	1.61 (0.91–2.85)		82	1.52 (1.07–2.15)
		Tertile 3	80	1.11 (0.79–1.57)		23	0.90 (0.47–1.71)		15	0.80 (0.39–1.64)		57	1.17 (0.78–1.76)
		P-trend*		0.50			0.77			0.70			0.35
	Lag 15 years												
		Tertile 1	92	1.00 (Ref)		23	1.00 (Ref)		23	1.00 (Ref)		69	1.00 (Ref)
		Tertile 2	82	1.27 (0.92–1.77)		11	0.69 (0.32–1.49)		28	1.45 (0.81–2.63)		71	1.46 (1.01–2.11)
		Tertile 3	94	1.12 (0.78–1.61)		27	0.86 (0.44–1.68)		18	0.80 (0.39–1.64)		67	1.21 (0.80–1.85)
		P-trend*		0.48			0.67			0.70			0.29
Duration of fire-exposed employment (n=4251)													
	No lag												
		Tertile 1	94	1.00 (Ref)		27	1.00 (Ref)		22	1.00 (Ref)		67	1.00 (Ref)
		Tertile 2	86	0.97 (0.72–1.30)		15	0.59 (0.31–1.12)		24	1.12 (0.63–2.00)		71	1.12 (0.80–1.57)
		Tertile 3	88	1.05 (0.78–1.42)		19	0.77 (0.42–1.42)		23	1.04 (0.58–1.90)		69	1.16 (0.82–1.64)
		P-trend*		0.77			0.35			0.88			0.40
	Lag 10 years												
		Tertile 1	88	1.00 (Ref)		25	1.00 (Ref)		18	1.00 (Ref)		63	1.00 (Ref)
		Tertile 2	85	1.00 (0.73–1.36)		14	0.63 (0.32–1.24)		28	1.50 (0.82–2.75)		71	1.14 (0.80–1.62)
		Tertile 3	95	1.07 (0.78–1.48)		22	0.72 (0.39–1.35)		23	1.13 (0.59–2.19)		73	1.22 (0.84–1.78)
		P-trend*		0.67			0.31			0.70			0.29
	Lag 15 years												
		Tertile 1	93	1.00 (Ref)		26	1.00 (Ref)		20	1.00 (Ref)		67	1.00 (Ref)
		Tertile 2	85	1.00 (0.73–1.36)		10	0.47 (0.22–1.00)		30	1.44 (0.80–2.59)		75	1.18 (0.83–1.68)
		Tertile 3	90	1.08 (0.77–1.51)		25	0.77 (0.41–1.45)		19	1.00 (0.49–2.01)		65	1.19 (0.80–1.78)
		P-trend*		0.69			0.42			0.90			0.36
Number of fires attended (n=4023)													
	No lag												
		Tertile 1	83	1.00 (Ref)		20	1.00 (Ref)		19	1.00 (Ref)		63	1.00 (Ref)
		Tertile 2	84	0.91 (0.67–1.24)		17	0.75 (0.39–1.45)		21	0.97 (0.52–1.80)		67	0.96 (0.68–1.36)
		Tertile 3	84	1.13 (0.83–1.53)		18	1.02 (0.54–1.93)		21	1.18 (0.63–2.20)		66	1.16 (0.82–1.64)
		P-trend*		0.45			0.98			0.60			0.40
	Lag 10 years												
		Tertile 1	83	1.00 (Ref)		21	1.00 (Ref)		19	1.00 (Ref)		62	1.00 (Ref)
		Tertile 2	84	0.93 (0.68–1.26)		14	0.58 (0.29–1.17)		21	0.98 (0.52–1.83)		70	1.04 (0.74–1.48)
		Tertile 3	84	1.12 (0.82–1.53)		20	0.99 (0.53–1.88)		21	1.18 (0.63–2.22)		64	1.16 (0.81–1.66)
		P-trend*		0.47			0.99			0.60			0.41
	Lag 15 years												
		Tertile 1	83	1.00 (Ref)		20	1.00 (Ref)		19	1.00 (Ref)		63	1.00 (Ref)
		Tertile 2	84	1.00 (0.73–1.37)		14	0.68 (0.33–1.38)		24	1.18 (0.64–2.17)		70	1.11 (0.78–1.57)
		Tertile 3	84	1.16 (0.84–1.59)		21	1.05 (0.54–2.01)		18	1.08 (0.56–2.10)		63	1.19 (0.82–1.71)
		P-trend*		0.37			0.84			0.81			0.36
Fire exposure score (n=4023)													
	No lag												
		Tertile 1	83	1.00 (Ref)		16	1.00 (Ref)		20	1.00 (Ref)		67	1.00 (Ref)
		Tertile 2	84	1.15 (0.84–1.56)		17	1.23 (0.61–2.48)		20	1.09 (0.58–2.05)		67	1.13 (0.80–1.59)
		Tertile 3	84	1.02 (0.72–1.44)		22	1.31 (0.63–2.72)		21	0.97 (0.49–1.93)		62	0.95 (0.64–1.40)
		P-trend*		0.86			0.47			0.96			0.85
	Lag 10 years												
		Tertile 1	84	1.00 (Ref)		17	1.00 (Ref)		21	1.00 (Ref)		67	1.00 (Ref)
		Tertile 2	83	1.03 (0.76–1.41)		17	1.08 (0.54–2.18)		20	0.93 (0.50–1.74)		66	1.02 (0.72–1.45)
		Tertile 3	84	0.99 (0.70–1.40)		21	1.11 (0.53–2.32)		20	0.86 (0.44–1.71)		63	0.96 (0.65–1.41)
		P-trend*		0.96			0.78			0.67			0.84
	Lag 15 years												
		Tertile 1	84	1.00 (Ref)		17	1.00 (Ref)		21	1.00 (Ref)		67	1.00 (Ref)
		Tertile 2	83	1.10 (0.80–1.51)		15	1.04 (0.50–2.17)		23	1.09 (0.59–2.00)		68	1.12 (0.78–1.59)
		Tertile 3	84	1.07 (0.75–1.52)		23	1.33 (0.63–2.78)		17	0.79 (0.39–1.61)		61	1.00 (0.67–1.49)
		P-trend*		0.68			0.45			0.56			0.95
Inhalation score (n=4023)													
	No lag												
		Tertile 1	85	1.00 (Ref)		18	1.00 (Ref)		17	1.00 (Ref)		67	1.00 (Ref)
		Tertile 2	90	1.06 (0.77–1.45)		20	1.07 (0.54–2.11)		25	1.45 (0.76–2.78)		70	1.05 (0.74–1.50)
		Tertile 3	76	1.10 (0.76–1.61)		17	0.95 (0.43–2.11)		19	1.31 (0.60–2.89)		59	1.16 (0.75–1.78)
		P-trend*		0.60			0.91			0.46			0.52
	Lag 10 years												
		Tertile 1	82	1.00 (Ref)		18	1.00 (Ref)		17	1.00 (Ref)		64	1.00 (Ref)
		Tertile 2	83	1.02 (0.74–1.40)		17	0.94 (0.47–1.91)		24	1.34 (0.70–2.58)		66	1.04 (0.72–1.49)
		Tertile 3	86	1.13 (0.78–1.64)		20	0.99 (0.46–2.16)		20	1.16 (0.54–2.52)		66	1.18 (0.77–1.80)
		P-trend*		0.52			0.98			0.68			0.46
	Lag 15 years												
		Tertile 1	86	1.00 (Ref)		18	1.00 (Ref)		18	1.00 (Ref)		68	1.00 (Ref)
		Tertile 2	88	1.09 (0.80–1.51)		19	1.14 (0.56–2.30)		25	1.38 (0.73–2.62)		69	1.08 (0.76–1.55)
		Tertile 3	77	1.12 (0.76–1.63)		18	0.98 (0.44–2.20)		18	1.26 (0.57–2.75)		59	1.16 (0.75–1.79)
		P-trend*		0.56			0.97			0.53			0.50
Diesel exhaust exposure score (n=3939)													
	No lag												
		Tertile 1	87	1.00 (Ref)		19	1.00 (Ref)		27	1.00 (Ref)		68	1.00 (Ref)
		Tertile 2	80	1.05 (0.77–1.42)		22	1.29 (0.70–2.39)		14	0.63 (0.33–1.20)		58	0.98 (0.69–1.39)
		Tertile 3	80	1.11 (0.81–1.54)		14	0.96 (0.46–1.99)		19	0.89 (0.47–1.69)		66	1.15 (0.80–1.66)
		P-trend*		0.52			0.99			0.59			0.47
	Lag 10 years												
		Tertile 1	82	1.00 (Ref)		17	1.00 (Ref)		27	1.00 (Ref)		65	1.00 (Ref)
		Tertile 2	83	1.06 (0.78–1.44)		22	1.38 (0.73–2.62)		15	0.62 (0.33–1.18)		61	0.98 (0.69–1.39)
		Tertile 3	82	1.22 (0.88–1.69)		16	1.13 (0.55–2.32)		18	0.86 (0.45–1.65)		66	1.25 (0.87–1.80)
		P-trend*		0.23			0.69			0.53			0.25
	Lag 15 years												
		Tertile 1	84	1.00 (Ref)		19	1.00 (Ref)		27	1.00 (Ref)		65	1.00 (Ref)
		Tertile 2	80	1.03 (0.75–1.41)		17	1.04 (0.53–2.02)		15	0.64 (0.33–1.21)		63	1.03 (0.72–1.46)
		Tertile 3	83	1.19 (0.86–1.65)		19	1.06 (0.53–2.10)		18	0.94 (0.49–1.80)		64	1.23 (0.85–1.78)
		P-trend*		0.30			0.87			0.72			0.28

^a^ International Classification of Diseases 10th revision code C61.

^b^ Aggressive prostate cancer defined as any of the following at diagnosis: summary stage reflecting regional lymph node or distant metastasis; TNM categories T4, N1 or M1 (from 1994); or a Gleason score ≥8 (from 2004).

^c^ Indolent prostate cancer defined as the following at diagnosis: local malignancy according to summary stage (from 1960) and TNM category (from 1994), and a Gleason score <8 (from 2004).

^d^ All prostate cancers that were not defined as aggressive (above).

* Modelled the ordinal variable as continuous to test for linear trend.

With number of fires attended, HR for the highest tertile was 1.13 (95% CI 0.83–1.53) for all PC combined, 1.02 (95% CI 0.54–1.93) for aggressive PC and 1.18 (95% CI 0.63–2.20) for indolent PC.

With fire exposure score, HR for the highest tertile was 1.02 (95% CI 0.72–1.44) for all PC combined, 1.31 (95% CI 0.63–2.72) for aggressive PC, and 0.97 (95% CI 0.49–1.93) for indolent PC. With inhalation score, HR for the highest tertile was 1.10 (95% CI 0.76–1.61) for all PC combined, 0.95 (95% CI 0.43–2.11) for aggressive PC, and 1.31 (95% CI 0.60–2.89) for indolent PC.

With diesel exhaust exposure score, HR for the highest tertile was 1.11 (95% CI 0.81–1.54) for all PC combined, 0.96 (95% CI 0.46–1.99) for aggressive PC, 0.89 (95% CI 0.47–1.69) for indolent PC, and 1.15 (95% CI 0.80–1.66) for unclassifiable PC.

Sensitivity analyses did not contribute to essential differences in results (results not shown).

**Figure 1:**
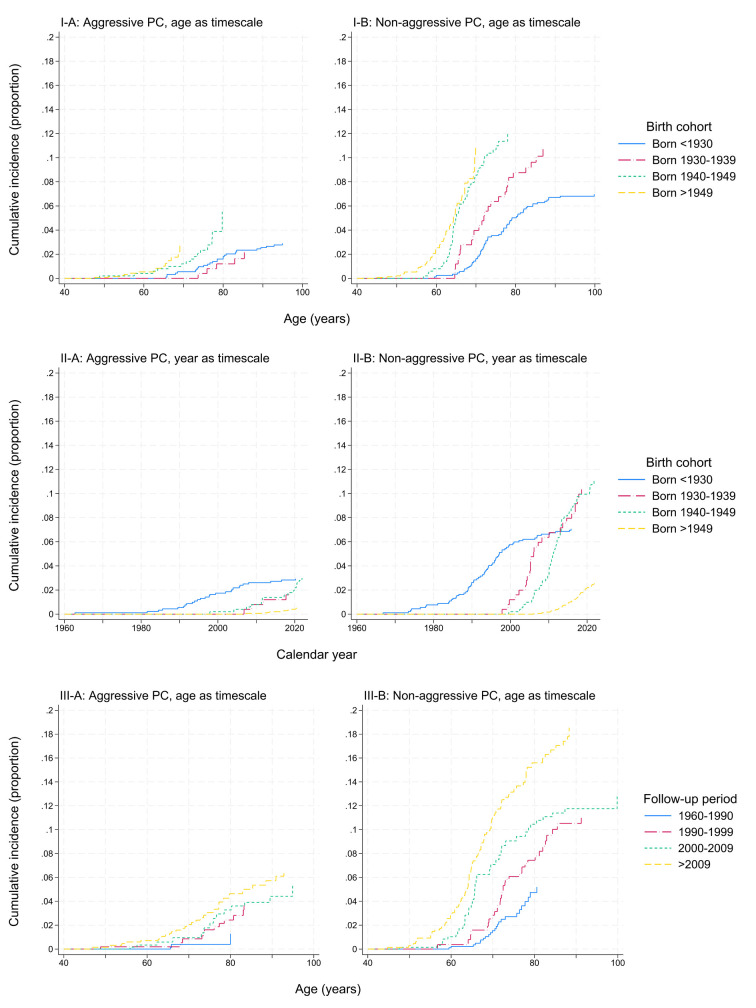
Cumulative incidence of A) aggressive and B) non-aggressive prostate cancer (PC) in the Norwegian Fire Departments Cohort (n=4251) with respect to I) age, by birth cohort; II) calendar year, by birth cohort; and III) age, by follow-up period.

The cumulative incidence of non-aggressive PC was higher than aggressive PC, and there was a shift to diagnoses at younger ages in later birth cohorts for both aggressive and non-aggressive PC (figure 1, I-A and I-B). For non-aggressive PC, the cumulative incidence for those born before 1930 at 80 years was 5.3%. For those born 1940–1949, the cumulative incidence surpassed 10% at 72 years; for those born after 1949, it surpassed 10% at 69 years.

With calendar year as the time scale (figure 1, II-A and II-B), for those before 1930, the cumulative incidence of non-aggressive PC increased from 2.8% in 1990 to 7.1% in 2015. For those born 1930–1939 and 1940–1949, the cumulative incidence of non-aggressive PC began to increase in 1997 and 1999, and surpassed 10% in 2018 and 2020, respectively.

When considering cumulative incidence by follow-up period (figure 1, III-A and III-B), there were only five cases of aggressive PC diagnosed 1960–1990. In the follow-up periods after 1990, the cumulative incidence of aggressive PC was 2.8–5.0% at 80 years. For non-aggressive PC, the cumulative incidence at age 80 increased from 5.2% in the period 1960–1990 to 15.9% in the period after 2009.

## Discussion

We investigated the associations between indicators of firefighting exposures and PC risk among 4251 men in the Norwegian Fire Departments Cohort, with analyses for overall, aggressive, and indolent PC. Our results provided little support for an association between our indicators of firefighting exposures and PC risk overall. Nonetheless, for aggressive PC, with few cases in each exposure tertile, a positive association was suggested with fire exposure score, which considered both the number of fires attended and the type of respiratory protective equipment used. For indolent PC and the combined group of non-aggressive PC (indolent plus unclassifiable), a weak positive association was suggested with number of fires attended and inhalation score.

Few studies have investigated PC in relation to specific exposures within firefighting previously, and findings have been mixed. In a US cohort study using the exposure indicators firefighting days, fire runs, and fire hours, no evidence of an exposure–response for PC incidence was observed (eg, HR 75^th^ versus 25^th^ percentile 1.02, 95% CI 0.91–1.14) (7). In contrast, an Australian study assessing cancer risk using the number and type of incidents attended observed increased PC risk with increasing number of fires attended [relative incidence ratio third tertile versus first 2.55 (95% CI 1.45–4.50)] (8).

Exposure at the fire scene predominantly entails exposure to fire smoke, comprised of incomplete combustion products such as PAH. Following biologically effective exposure, PAH are hypothesized to initiate or contribute to prostate carcinogenesis through the formation of PAH-DNA adducts, which can be genotoxic and lead to DNA replication errors (27). Barul & Parent (28) observed an OR of 1.37 (95% CI 0.65–2.89) for aggressive PC for those ever exposure to PAH from wood compared to those never occupationally exposed, with this exposure mostly occurring in firefighting occupations (28). However, Boers et al (29) found no evidence of an association between occupational PAH exposure and PC risk.

Our study spanned several decades of considerable changes in working conditions (17). Our indicator fire exposure score considered both fire statistics and the type and use of PPE. This indicator, like others, was time-dependent in the analyses, with exposure accruing according to each individual’s position, department, and calendar years of employment. Nonetheless, firefighters’ occupational exposures remain difficult to characterize and quantify, largely due to the limited historical data on exposures and exposure proxies, and it is difficult to assess how the potential varying validity of the indicators have impacted on our findings.

Firefighters can also be exposed to diesel exhaust, which comprises a complex mixture of particles, PAH, and volatile organic compounds, among others (30). In 1998, Seidler et al (31) examined the association between occupational diesel exhaust exposure and PC, and found increased PC risk among those exposed to over 25 dose-years versus those never exposed (OR 3.7, 95% CI 1.4–9.8). However, more recent studies examining this association have reported no association (29, 32).

Night shift work has been classified as a probable prostate carcinogen (33), but the extent to which firefighters experience night shift work in the absence of fires and emergencies is unclear. Heat stress has also been investigated for its potential role in PC risk as it displays some key characteristics of human carcinogens (34, 35). However, a pooled case–control study examining heat exposure and PC risk found no evidence of an association (35).

In addition, PC incidence has been influenced by widespread opportunistic PSA testing, demonstrated by the steep increase in incidence observed in Norway from the 1990s marking introduction of the PSA test and the beginning of the “PSA era” (9–11). For the general Norwegian population in 1960–1993, mean age at diagnosis was 72.5 years; in 2007–2017, it was 69.4 years. Among firefighters identified through census data, mean age at diagnosis went from 70.4 to 65.1 years for the same periods (10, 12).

The cumulative incidence of PC has been examined to elucidate the effect of PSA testing in Norway. Valberg et al (9) found that among birth cohorts entering the PSA era at increasingly younger ages, a higher proportion of individuals were being diagnosed with PC at younger ages compared to the pre-PSA era (9). In our study cohort, we observed a rise in cumulative incidence of non-aggressive PC at younger ages in later birth cohorts and more recent follow-up periods, while for aggressive PC, this tendency was less marked. Although there may be other explanatory factors such as changes in background or occupational exposures, we expect that these trends are predominantly explained by an increasing influence of opportunistic PSA testing beginning at younger ages after 1990.

We examined PC risk and cumulative incidence by clinical stage, with the expectation that PSA testing has had a greater influence on diagnosis of non-aggressive PC. However, aggressive versus non-aggressive PC is not independent, as a non-aggressive PC diagnosis is a competing event that precludes a subsequent aggressive primary PC diagnosis. At present, we do not (and cannot) know the proportion of non-aggressive PC detected early through opportunistic PSA testing that would have progressed to aggressive PC to be detected otherwise.

In the general population, Valberg et al (9) also observed a decline in age-specific PC rates at older ages and that the cumulative incidence across birth cohorts did not differ, suggesting “greater depletion of a limited pool of susceptible individuals” (9). If the “susceptible pool” in the general population is already largely depleted, it may be hypothesized that among firefighters, it is not only a heightened effect of PSA testing contributing to elevated PC incidence. Rather, it may also be that the “susceptible pool” has been enlarged, possibly due to an occupational exposure. This explanation could be in-line with findings of elevated PC incidence among firefighters compared to the general population. However, no studies conducting internal comparisons with more detailed exposure metrics, including ours, have consistently identified an occupational exposure associated with PC among firefighters nor any other occupational group thus far.

### Strengths and limitations

A strength of our study is the use of various indicators of firefighting exposures, developed in cooperation with our project reference group. These indicators considered department-specific fire statistics and working conditions that likely influenced occupational exposures, namely changes in PPE and the use and quality of SCBA. This should have contributed to greater differentiation in exposure estimates. However, many in our cohort had long employment and exposure durations, which limited contrasts between exposure tertiles and would have resulted in an insufficient number of cases if a less exposed reference group was chosen. Further, like other studies using exposure indicators, we had no exposure measurements to include in nor to validate our indicators, and it is possible that misclassification has occurred. Indicators were developed and applied without knowledge of health status; therefore, potential misclassification would probably be non-differential (36). Assuming non-differential, non-systematic errors, misclassification would be expected to attenuate the HR for the highest exposure category in our models (37).

Given the population size and limited number of firefighters in Norway, our study sample is relatively large. The 15 participating departments provided firefighting services for nearly 50% of the Norwegian population as of 2019 (17). However, our cohort was based on employment records, and data on lifestyle habits and non-firefighting employment history was not available. As firefighters are required to be in good health to become and remain employed, potential bias from a HWSE is often of concern in studies of this occupational group. However, most occupational groups are relatively homogeneous, and confounding from uncontrolled lifestyle factors is therefore expected to be small in internal comparisons (36).

With the high degree of coverage and strict quality control measures at the CRN (38), we expect that the cancer incidence data were valid, and the data provided a valuable opportunity for analysis by clinical characteristics. Although follow-up began in 1960, most PC were diagnosed in 1994 or later, when TNM staging was included. Still, a large proportion of PC were unclassifiable, and the number of cases for analysis according to clinical stage was limited. In particular, the definition of indolent PC (21) likely contributed to the high number of unclassifiable PC. Our results for the broader group of non-aggressive PC, including both indolent and unclassifiable PC with the same overall proportion diagnosed in 1994 or later, provided somewhat more precise estimates for a group of PC that we expect were predominantly diagnosed early without distant metastasis (22).

### Concluding remarks

In our study of PC risk among firefighters, we found little support for an association between firefighting exposures and PC risk. However, our study had few cases in analyses by clinical stage. Data demonstrated elevated incidence in periods with elevated diagnostic opportunity from PSA testing, but the magnitude of a medical surveillance bias remains unclear. Studies of PC risk among firefighters also remain challenged by difficulties in exposure characterization and a limited understanding of PC etiology.
